# Facial Appearance Dissatisfaction Explains Differences in Zoom Fatigue

**DOI:** 10.1089/cyber.2021.0112

**Published:** 2022-02-10

**Authors:** Rabindra Ratan, Dave B. Miller, Jeremy N. Bailenson

**Affiliations:** ^1^ Department of Media & Information, Michigan State University, East Lansing, Michigan, USA.; ^2^ Department of Industrial Engineering and Management Systems, University of Central Florida, Orlando, Florida, USA.; ^3^ Department of Communication, Stanford University, Stanford, California, USA.

**Keywords:** appearance dissatisfaction, survey, videoconferencing, virtual meeting fatigue, Zoom fatigue

## Abstract

Viewing self-video during videoconferences potentially causes negative self-focused attention that contributes to virtual meeting (VM) or “Zoom” fatigue. The present research examines this proposition, focusing on facial dissatisfaction—feeling unhappy about one's own facial appearance—as a potential psychological mechanism of VM fatigue. A study of survey responses from a panel of 613 adults found that VM fatigue was 14.9 percent higher for women than for men, and 11.1 percent higher for Asian than for White participants. These gender and race/ethnicity differences were found to be mediated by facial dissatisfaction. This study replicates earlier VM fatigue research, extends the theoretical understanding of facial dissatisfaction as a psychological mechanism of VM fatigue, and suggests that practical approaches to mitigating VM fatigue could include implementing technological features that reduce self-focused attention during VMs (e.g., employing avatars).

Given the prevalence of remote work both during and likely beyond the COVID-19 pandemic,^1–4^ it is important to understand and address the exhaustion that occurs after long periods of videoconferences, referred to as “Zoom fatigue,” videoconferencing fatigue, or virtual meeting (VM) fatigue. VM fatigue has been identified as a detriment to worker well-being and productivity^5,6^ and is theorized to result from multiple factors, including increased cognitive load due to prolonged gaze from others, the apparent closeness of others, and reduced mobility^7^; unmet expectations regarding synchrony and nonverbal cues^8^; and the loss of a sense of place, lessened scaffolding and supervision, and reduced dynamic and nonconscious distribution of work among teammates.^9^ This study builds on research suggesting that viewing self-video causes mirror anxiety—negative self-focused attention—which is psychologically taxing and contributes to VM fatigue.^7,10^ Supporting this reasoning and highlighting the importance of this topic, a 4-week field experiment found that VM fatigue was higher for participants randomly assigned to keep their cameras on (compared with off) during VMs and that VM fatigue fully mediated a negative effect of camera condition on worker voice and engagement during meetings.^6^ We extend this line of inquiry to examine one factor that potentially explains why some people experience mirror anxiety and thus VM fatigue: facial appearance dissatisfaction (or simply *facial dissatisfaction*).^11^ For individuals with higher levels of facial dissatisfaction, viewing self-video likely causes more negative self-focused attention and thereby increases VM fatigue.

There is evidence that increases in videoconferencing use during the COVID-19 pandemic were associated with greater appearance dissatisfaction, especially facial dissatisfaction, as VM systems usually display faces prominently. Cosmetic surgeons and dentists reported receiving a higher number of requests from people interested in improving their appearance because of the time they were spending in videoconferencing.^12–14^ Furthermore, studies have found that viewing self-video contributes to facial dissatisfaction, especially when individuals feel self-objectified.^15,16^ These patterns suggest that facial dissatisfaction is a manifestation of psychological distress that contributes to VM fatigue.

This study examines facial dissatisfaction as a facet of negative self-focused attention that may help explain differences in VM fatigue between social groups. Many cultures promote gendered beauty norms and “whiteness”^17–21^ that pressure women and people of color (POC) to conform with these norms in their appearance, especially in the workplace.^22–25^ These pressures on women and POC potentially contribute to higher levels of facial dissatisfaction, which when activated by prolonged viewing of self-video may then lead to VM fatigue. One survey-based study found that women report more VM fatigue than men, that differences in mirror anxiety mediate this gender difference, and that Whites experience less VM fatigue than people of other races (although with a small effect size).^10^ Similarly, a field experiment found that the increase in VM fatigue induced by having the VM camera on (compared with off) was higher for women than for men, as well as for lower tenure workers, supporting the reasoning that social group differences in negative self-focused attention are responsible for VM fatigue.^6^

Extending these previous studies, this research aims to replicate previous findings of gender and race/ethnicity differences in VM fatigue,^10^ while also considering facial dissatisfaction as a mediator of these differences (e.g., a path from gender to facial dissatisfaction to VM fatigue). Hence, we hypothesize the following:

H1: VM fatigue is higher for women than for men.

H2: VM fatigue differs by race/ethnicity and, specifically, is lower for people who identify as White than who identify as (a) Black/African American, (b) Latino/Hispanic, and (c) Asian.

H3: VM fatigue is positively associated with facial dissatisfaction.

H4: The effects of gender and race on VM fatigue are mediated by facial dissatisfaction.

## Method

The survey panel platform Prolific was used to recruit participants who report living in the United States, working from home, and having previously completed at least two VMs using Zoom on the same day as they completed the survey. Zoom, recognized as the most widely used VM application with >200 million daily users,^26^ was specified in the IRB-approved survey to maintain platform consistency in participant experiences. Of the 798 people who accessed the survey, 179 did not complete >25 percent of the survey (including the VM fatigue measure) and were not considered participants. Of the remaining 619 participants, 6 were missing race data and thus were not included in any analyses. Another four did not complete the facial dissatisfaction measure, so they were not included in analyses that involved this variable. In other words, the final *N* was 613, but only 609 could be included in analyses that involved facial dissatisfaction.

According to Prolific's demographic data, reported age (*n* = 605) ranged from 18 to 68 (*M* = 30.89, *SD* = 8.83). The sample was intentionally stratified for gender (*n* = 613; 51.2 percent women and 48.8 percent men) and race (*n* = 613), with at least 130 participants included from each of the four race/ethnicity groups that are most broadly represented in the United States^27^: Black/African American (*n* = 132), Latino/Hispanic (*n* = 152), Asian (*n* = 164), and White (*n* = 165). Other race/ethnicity groups were not included due to their low representation in the Prolific survey panel population.

### Measures

*Virtual Meeting Fatigue,* the primary dependent variable of interest, was measured with the Zoom Exhaustion & Fatigue Scale (ZEF Scale), which has been validated through correlations with other relevant VM attributes (i.e., negative attitude, meeting duration, and frequency).^28^ This scale includes 15 items split evenly across 5 reliable subfactors: general fatigue (e.g., “How tired do you feel after videoconferencing?”), visual fatigue (“How blurred does your vision get after videoconferencing?”), social fatigue (“How much do you tend to avoid social situations after videoconferencing?”), motivational fatigue (“How much do you dread having to do things after videoconferencing?”), and emotional fatigue (“How emotionally drained do you feel after videoconferencing?”). A single ZEF composite mean score metric was calculated from 5-point Likert-type scale responses of agreement across items (*M* = 2.60, *SD* = 0.92, α = 0.95, skewness = 0.29) with higher values reflecting more VM fatigue.

*Facial dissatisfaction* was measured using items from a subconstruct of a larger validated Negative Physical Self Scale.^11^ This trait-level measure includes 11 items, including “I am depressed about how my face looks,” “If it is possible, I will change the way my face looks,” and “I do not like what I see when I look in the mirror.” A mean score across the 5-point Likert-type scale responses was used to create a single facial dissatisfaction metric with acceptable reliability (*M* = 1.78, *SD* = 0.78, α = 0.92; skewness = 1.34). To address the non-normality, we performed the common procedure of a natural logarithmic transformation on this variable.^29^ The resulting transformed variable displayed acceptable skewness (0.58) and was used in the analyses.

## Results

**FIG. 1.**
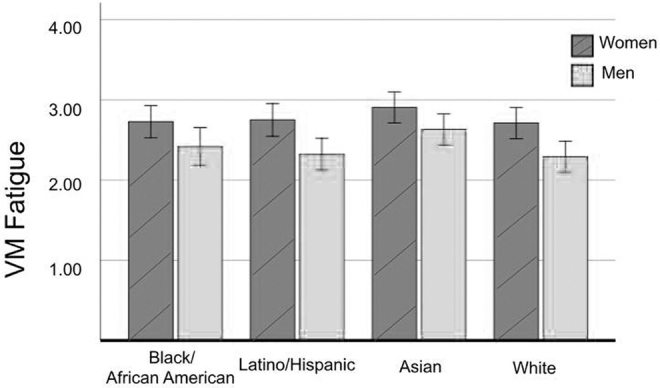
VM fatigue by gender and race/ethnicity. VM, virtual meeting.

An analysis of variance test was conducted with the 15-item ZEF measure of VM fatigue as the dependent variable, and participant gender (man, woman), race/ethnicity (four categories), and the interaction between the two as categorical predictors (Fig. 1 and Table 1). VM fatigue was found to be 14.9 percent higher for women (*M* = 2.77, *SE* = 0.05) than for men (*M* = 2.41, *SE* = 0.05), *F*(1, 608) = 25.19, *p* < 0.001, η_p_^2^ = 0.04, providing support for H1. There was a significant, although small, difference by race/ethnicity, *F*(1, 608) = 3.08, *p* = 0.03, η_p_^2^ = 0.015, and no significant difference for their interaction term, *F*(3, 608) = 0.37, *p* = 0.77. *Post hoc* comparisons of race/ethnicity groups suggested that VM fatigue was 11.1 percent higher for Asian (*M* = 2.77, *SE* = 0.07) than for White (*M* = 2.50, *SE* = 0.07) participants according to Tukey's HSD protected test [lower limit confidence interval (LLCI) = 0.0207, upper limit confidence interval (ULCI) = 0.5274, *p* = 0.03], with no other significant *post hoc* differences found. These results provide support for H2c, although with a small effect size, but no support for H2a or H2b.

**Table 1.** Virtual Meeting Fatigue by Race/Ethnicity and Gender

	Mean	SD
*Black/African American*		
Women	2.73	0.90
Men	2.42	0.85
Latino/Hispanic		
Women	2.75	1.03
Men	2.29	0.88
Asian		
Women	2.91	0.97
Men	2.63	0.90
White		
Women	2.71	0.80
Men	2.29	0.76

**FIG. 2.**
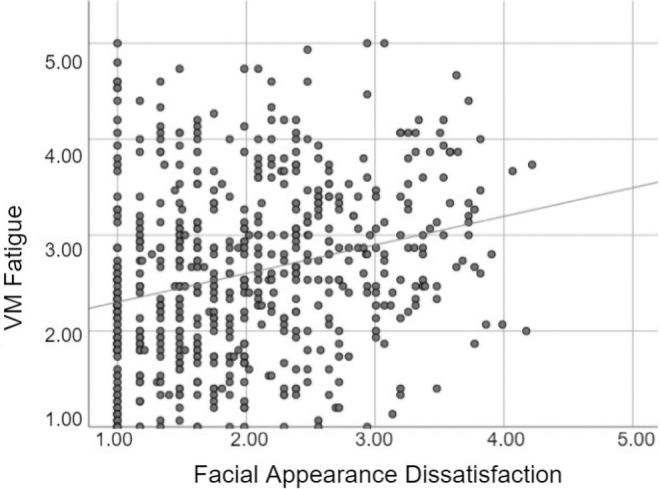
VM fatigue by facial appearance dissatisfaction.

A one-tailed Pearson's correlation test found that VM fatigue and facial dissatisfaction were positively correlated (*r* = 0.26) (see Fig. 2 for scatterplot). To confirm that this association was independent of the gender and race/ethnicity effects found in the previous test, we conducted a stepwise regression with participant gender (man, woman), race (Asian or White, given this was the only significant difference found from the comparisons of racial/ethnic groups), and facial dissatisfaction as the predictors of VM fatigue. Results (Table 2) suggest that facial dissatisfaction indeed predicted VM fatigue independently and in fact contributed most to the *R*^2^ value.

**Table 2.** Stepwise Regression Predicting Virtual Meeting Fatigue

	Model 1			Model 2			Model 3		
	β	*t*	*p*	β	*t*	*p*	β	*t*	*p*
Gender	−0.20	−3.61	^***^	−0.20	−3.61	^***^	−0.16	−3.10	^**^
Race (Asian vs. White)				−0.15	−2.83	^***^	−0.11	−2.20	^**^
Facial dissatisfaction							0.30	5.83	^***^
*df*	325			325			325		
Adj. *R*^2^	0.036			0.056			0.144		

^*^ *p* < 0.05, ^**^p < 0.01, ^***^p < 0.001.

An ordinary least squares regression path analysis (Hayes PROCESS,^30^ Model 4, 10,000 bootstrapped samples)—with standardized VM fatigue as the outcome, participant gender (woman or man) as the predictor, standardized facial dissatisfaction as the mediator, and participant race (Asian or White) as a covariate—found a significant indirect effect of being a woman compared with being a man on VM fatigue through facial dissatisfaction (β = −0.0662, LLCI = −0.1370, ULCI = −0.0043, *R*^2^ = 0.1514). A second path analysis—with standardized VM fatigue as the outcome, participant race (Asian or White) as the predictor, standardized facial dissatisfaction as the mediator, and participant gender as a covariate—also found a significant indirect effect of being Asian compared with being White on VM fatigue through facial dissatisfaction (β = −0.0752, LLCI = −0.1457, ULCI = −0.0129, *R*^2^ = 0.1514). Together, two models provide support for H4, suggesting that facial dissatisfaction mediates the influence of gender (women compared with men) and race/ethnicity (for Asian compared with White) participants on VM fatigue.

## Discussion

This study examines the role of facial appearance dissatisfaction in VM fatigue. Survey responses from a panel of adults suggest that VM fatigue is associated with facial dissatisfaction, which was found to mediate VM fatigue differences by gender and race/ethnicity. The results replicate previous findings that VM fatigue is higher among women than among men.^6,10^ Furthermore, Asian participants reported more VM fatigue than White participants, although with a small effect size, but no other significant race/ethnicity differences were found. These results extend VM fatigue research to consider facial dissatisfaction as a mechanism, which aligns with the theoretical proposition that viewing self-video for long periods is psychologically detrimental.^7^

Together, the findings—that VM fatigue was higher for women than for men and for Asian than for White participants (although with small effect sizes), and that these differences were mediated by facial dissatisfaction—support the reasoning that VM fatigue is caused at least, in part, by the harmful psychological load (i.e., negative self-focused attention) induced through viewing of self-video.^6,7^ The finding is consistent with a survey-based study that found that gender differences in VM fatigue were mediated by mirror anxiety,^10^ as well as a field experiment that found that the increase in VM fatigue from having the camera on (compared with off) is higher for women than for men.^6^ Women are more likely to experience facial dissatisfaction than men, which makes them more likely to experience mirror anxiety and thus VM fatigue. This study did not measure mirror anxiety, so future research should explicitly test this expanded path model. Furthermore, these constructs, facial dissatisfaction and mirror anxiety, may both relate to more general psychological aspects of negative self-focused attention for which gender differences have also been found, such as self-objectification (with connections to body shame)^31,32^ and upward social comparison (especially gendered in Western cultures).^33–35^ Similarly, Asians have been found to express higher levels of dissatisfaction with facial features (e.g., eye shape, skin smoothness/whiteness) than Whites,^36–39^ which supports the reasoning that viewing self-video in VMs might increase focus on facial dissatisfaction, especially for Asians, and thereby increase VM fatigue.

### Practical implications

These findings support the need for VM platforms to include features that mitigate facial dissatisfaction, mirror anxiety, or negative self-focused attention. Hiding self-video altogether might be one solution,^6,7^ but this potentially causes other problems, such as a lack of awareness about self-presentation or even potential unintended broadcasts (e.g., embarrassing background activity). Self-video effects, such as face smoothing^40^ and filters that slightly modify personal appearance (e.g., digital hats),^41^ might help, but these effects might not reduce self-focused attention sufficiently if they only enhance a small portion of the user's self video, and further, may adversely change cultural beauty standards if the practice becomes common. Fully digital avatars, which are increasingly easily integrated across media platforms from video games to VMs,^42,43^ can be used to occlude video of the entire user and thus help reduce negative self-focused attention while still allowing the user to self-monitor while interacting with others both verbally and nonverbally in a VM. Furthermore, avatars can be designed to transform social interactions and influence user behaviors in positive ways.^44–46^ Future VM research should consider using such avatar attributes to address the inevitable challenges of our increasingly remote workforce.

### Limitations and conclusions

Notable limitations of this study include the focus on only four race/ethnicity categories, the treatment of Asians as a single group (combining south and east Asians), and the lack of specific measures related to different types of beauty standards, which vary between racial/ethnic groups.^47^ Further, there were fewer Black/African American (132) and Latino/Hispanic (152) participants than Asian (164) and White (165) participants, despite the attempt to evenly stratify the sample, likely due to lower number of potential participants on the Prolific platform from these populations. We should note that the present analytic methods are sufficiently powered and robust to unequal sample sizes—especially given that all groups had 132 or more participants.^48^ Still, future research on this topic would be well served by larger scale data collections that include a wider range of races/ethnicities with larger samples within each group as well as measures that are appropriate in consideration of differing beauty standards between groups.

A further limitation relates to the treatment of facial dissatisfaction as a trait, despite the likelihood that appearance dissatisfaction likely depends on contextual factors (e.g., use of makeup and clothing). Although we would expect that such state-level differences would randomize out across large groups and thus would not pose a threat to the present study's internal validity, future research should still control for or otherwise consider contextual factors that influence appearance dissatisfaction to extend an understanding of contributors to VM fatigue. Internal validity is also limited by the survey-based methodology. Future research should use methodologies that afford more control (e.g., field experiments) to confirm and extend the present findings.

The limitations of this study notwithstanding, this exploratory research is an early step toward understanding and reducing VM fatigue. This is an important social issue given that VMs raise further obstacles to equity and inclusion, similar to those found in face-to-face team meetings,^16,49–52^ such as unequal talking time and emphasis on personal appearance for women and POC,^22–24,53–56^ and VMs will likely remain an important component of remote work.^1–4^
